# A scoping review of methods for assessment of sex differentials in early childhood mortality

**DOI:** 10.1186/s12887-021-02503-8

**Published:** 2021-01-26

**Authors:** Janaína Calu Costa, Cesar G. Victora

**Affiliations:** International Center for Equity in Health, Postgraduate Program in Epidemiology, Federal University of Pelotas. Marechal Deodoro, 1160, 3rd floor, Pelotas, 96020-220 Brazil

**Keywords:** Gender bias, Excess female mortality, Under-five mortality, Epidemiology, Review

## Abstract

**Background:**

While assessment of sex differentials in child mortality is straightforward, their interpretation must consider that, in the absence of gender bias, boys are more likely to die than girls. The expected differences are also influenced by levels and causes of death. However, there is no standard approach for determining expected sex differences.

**Methods:**

We performed a scoping review of studies on sex differentials in under-five mortality, using PubMed, Web of Science, and Scopus databases. Publication characteristics were described, and studies were grouped according to their methodology.

**Results:**

From the 17,693 references initially retrieved we included 154 studies published since 1929. Indian, Bangladeshi, and Chinese populations were the focus of 44% of the works, and most studies addressed infant mortality. Fourteen publications were classified as *reference* studies, as these aimed to estimate expected sex differentials based upon the demographic experience of selected populations, either considered as gender-neutral or not. These studies used a variety of methods – from simple averages to sophisticated modeling – to define values against which observed estimates could be compared. The 21 *comparative* studies mostly used life tables from European populations as standard for expected values, but also relied on groups without assuming those values as expected, otherwise, just as comparison parameters. The remaining 119 studies were categorized as *narrative* and did not use reference values, being limited to reporting observed sex-specific estimates or used a variety of statistical models, and in general, did not account for mortality levels.

**Conclusion:**

Studies aimed at identifying sex differentials in child mortality should consider overall mortality levels, and report on more than one age group. The comparison of results with one or more reference values, and the use of statistical testing, are strongly recommended. Time trends analyses will help understand changes in population characteristics and interpret findings from a historical perspective.

**Supplementary Information:**

The online version contains supplementary material available at 10.1186/s12887-021-02503-8.

## Key findings and recommendations

- There is no standard method for assessing sex differentials in under-five mortality in order to identify unexpected sex ratios that may suggest the presence of gender bias.
- The identification of unexpected sex ratios will vary depending on the assumptions and methods used to define expected values.
- The historical demographic experiences of currently high-income countries provide the main reference parameters for the assessment of sex differentials, but different epidemiological profiles are present in current-day populations.
- Most studies failed to consider the overall level of mortality, relied upon a single expected value for multiple countries, and did not report on statistical assessments of the differences between observed and expected estimates.
- The use of external parameters for comparison that consider the level of overall mortality and apply appropriate statistical methods to compare observed and expected ratios are recommended.
- Whereas gender bias against girls is the most frequent explanation for unexpected sex ratios, methods should also consider the possibility of higher-than-expected mortality of boys.

## Background

Mortality is a result of the interaction between several complex factors such as genetics, environment, health care, and behaviors, and there is overwhelming evidence that male children have a higher risk of death than do females [1–3]. Nevertheless, there are many historical examples of excess female mortality at specific periods of the life course from childhood through adulthood [4–7].

Due to biological characteristics, female newborns and infants have an advantage in survival and enjoy lesser vulnerability to mortality due to perinatal conditions, congenital anomalies, and infectious diseases [8–10]. Beyond the first year of life, girls do not present the same advantage concerning certain infectious diseases, which are the primary causes of death from 1 to 4 years of age, and this advantage remains but tends to decline [1, 2]. Even so, in circumstances in which boys and girls receive the same care, higher mortality among male children is observed and equality in male and female death rates (or higher female mortality) may be evidence of inequity [11].

Excess female mortality may be an indicator of influences that outweigh the biological survival advantages of girls [1]. For instance, in some populations, the female advantage can be eroded if girls are deprived of access to health care or proper nutrition, suggesting discriminatory behaviors and gender bias in child care due to community male preference [1, 12].

Although sex differentials in early childhood mortality are recognized as important issues globally, there is no consensus about what would be expected in the absence of discrimination. A further challenge is that the magnitude of differences will vary according to total mortality levels and to the distribution of causes of death [8, 11, 13].

Many studies have assessed differences in mortality between boys and girls and sets of reference values for the relationship between the sex-specific estimates have been proposed, however, there is not a standard method for such analyses. In particular, the literature still lacks a proper description of how the estimates for such variations had been defined. By bringing together the approaches used so far and using a scoping review methodology, we described how studies addressed sex differentials in early childhood mortality with a special focus on the methods used to measure such discrepancies.

## Methods

### Data sources and search strategy

**Fig. 1:**
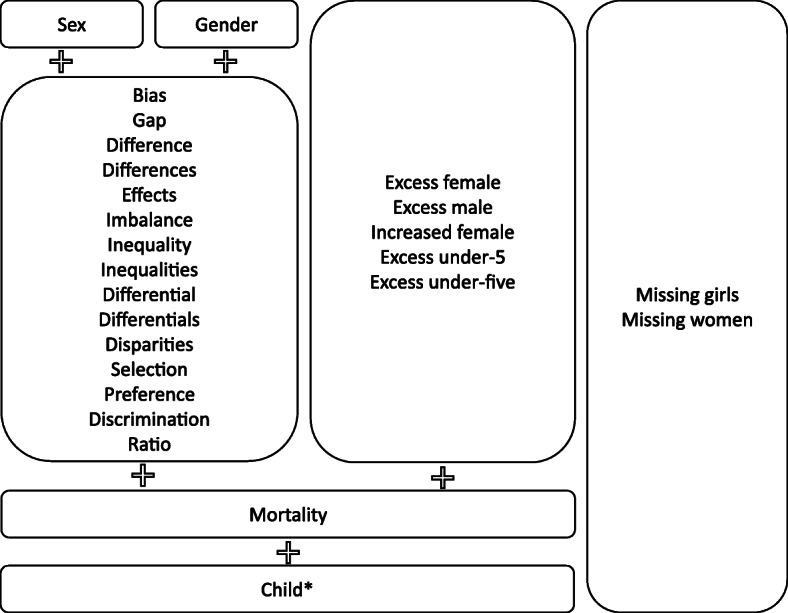
Search strategy

We conducted searches on PubMed, Web of Science, and Scopus electronic databases for potentially relevant publications on sex differentials in mortality among children, with no date limits or language restrictions. The searches included combinations of the key terms “gender” and “sex” with each of the selected words that reflect differences between the groups, namely “bias”, “gap”, “difference”, “effects”, “imbalance”, “inequality”, “differential”, “disparities”, “selection”, “preference”, “discrimination”, and “ratio”, plus the words “mortality” and “child*”. Additionally, we undertook searches using commonly used terms for excessive deaths: “excess male”, “excess female”, “increased female”, “excess under-5” and “excess under-five”, combined with “mortality” and “child*”, as well as “missing girls” and “missing women”. The list of terms and the full search strategy are presented in Fig. 1.

After removing the duplicates, we reviewed titles and abstracts for inclusion and if any doubts persisted, the full publication was read and included if it met the eligibility criteria. Finally, the reference lists of selected studies were searched for further relevant works. The search ended in July 2020.

### Eligibility criteria

Studies were eligible for inclusion if they quantitatively explored and discussed differentials in early child mortality by sex. These publications include any indicator of deaths of children under the age of five, videlicet, neonatal (NMR, 0–28 days of life), post-neonatal (PNMR, 28 days to 11 months and 29 days), infant (IMR, 0–11 months), child (CMR, 1–4 years), and under-five mortality rate (U5MR, 0–4 years), or any publication-specific age range. We excluded studies focusing on specific diseases, conditions (e.g. preterm), or behaviors (e.g. accidents and injuries), and those that only described differences in causes of death but did not report mortality levels. Also, we did not include works that addressed fetal mortality or actual population sex ratios as a measure of differential survival by sex.

### Article relevance screening

For each selected reference, we extracted publication characteristics, methodology, and results of interest. Our main focuses were the methods used to measure differences in mortality between boys and girls, including the data sources used to estimate mortality and to define the comparison reference (when applicable), statistical methods for comparing sex-specific estimates and observed and expected estimates. Despite some differences with the methodology used for systematic reviews, we present the relevant information in a PRISMA checklist (Supplementary Table S1).

### Studies characterization

We defined three categories of publications based on similarities in the methodological approach used in each of them.

The first category was labeled as ‘*Reference’* and include those studies that proposed specific reference populations as parameters of comparison (e.g. a set of high-income countries, populations for which gender discrimination is assumed to be absent, or the countries from a given region) and applied well-defined methods to estimate the expected values for sex differentials in childhood mortality. The author then used the results obtained from the reference population to compare with sex-specific estimates observed in other populations.

The second category consists of ‘*Comparative’* studies, which were those that reported a comparison of data from a specific population to a previously defined expected estimate for sex differentials mortality (e.g., published sex ratios for a given reference population) or simply comparing results from different geographies in order to describe their similarities and differences without assuming one of them as expected parameter.

The main difference between the *reference* and the *comparative* studies is that the former present a specific methodology for calculating the differential survival for boys and girls in the reference population defining expected values, while the latter group applies the methodology presented in a reference study, or else only compare results from two or more populations.

Finally, the third group includes the ‘*Narrative’* studies*,* that is, those in which the authors did not use any reference or comparison standard, and simply described sex differentials in mortality based only on their data.

### Additional information

Studies included in the *reference* group were also identified as either *descriptive* or *prescriptive*, based on a previous classification [14]. The former group includes studies that show how sex differentials in mortality vary, regardless of whether gender bias is assumed to exist, that is, without an a priori judgment. The latter group consists of publications in which mortality rates by sex are compared to values that would be expected in societies where gender discrimination is believed to be low or absent. Also, the number of citations for each *reference* study was collected from Web of Science metrics.

Based on the titles we generated a word cloud, a visual representation of text data in which the items are weighted according to their frequency. It helps us to visualize how the terms have been used in the field.

## Results

### Selection of studies

**Fig. 2:**
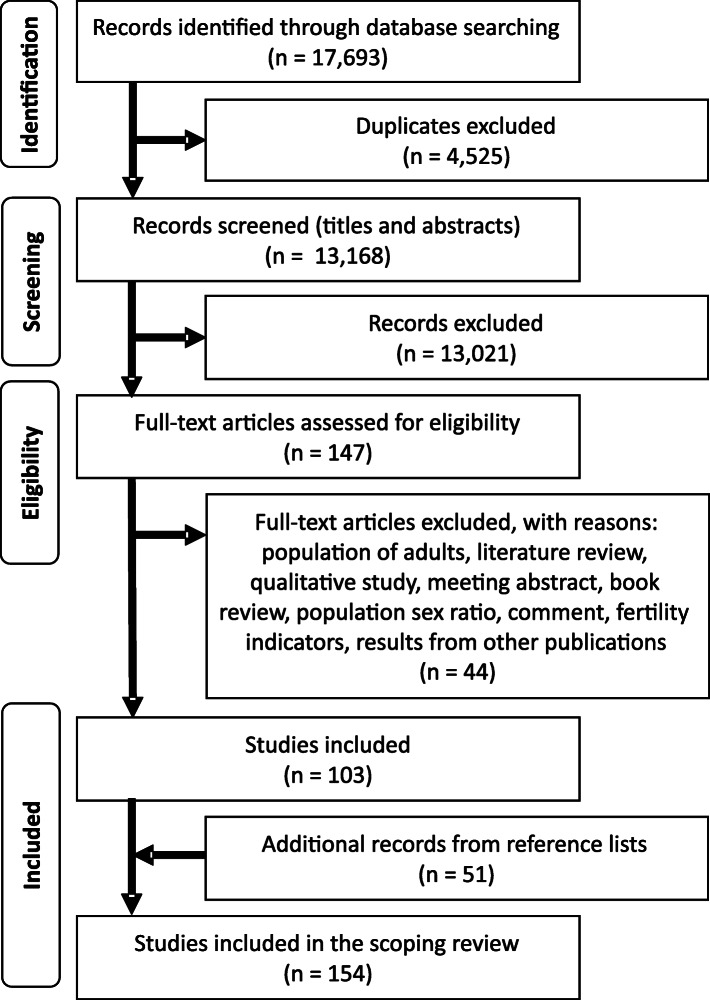
Study selection flow diagram

The initial searches yielded 17,693 publications. After removing the 4525 duplicates, 13,168 titles and abstracts were screened, and afterward, 147 articles were assessed in detail. Of these, 103 met all the inclusion criteria. From the lists of references, we found and could get access to other 51 works, resulting in 154 studies included in this review. The flow diagram displays the search strategy and study selection process (Fig. 2).

### General characteristics of studies included

The word cloud based on titles revealed a diversity of terminologies used in the publications, especially the possible interchangeable use of “sex” and “gender” and the high frequency of studies from India (Supplementary Figure S1).

The period of mortality estimates ranged from 1700 to 2016, which may refer to the survey year, the period before the survey for which the rates were estimated, or the actual years when the deaths occurred. Many studies assessed changes over time, either by comparing two points in time or by analyzing time series with several points [1–3, 5–7, 11, 13, 15–64].

The studies were published in a period between 1929 and 2020. Up to the 1980s, there were only thirteen publications [15, 27, 51, 54, 56, 57, 61, 63–68], and the number increased to 21 in the 1980s [8, 23, 28, 34, 58, 59, 69–83], 46 in the 1990s [3, 4, 6, 12, 22, 24, 33, 39, 43, 44, 46, 49, 52, 53, 84–115], decreased to 30 in the 2000s [7, 11, 16, 17, 21, 25, 26, 36, 41, 47, 50, 60, 62, 116–132], and reached 44 in the period between 2010 and 2020 [1, 2, 5, 13, 14, 18–20, 29–32, 35, 37, 38, 40, 42, 45, 48, 55, 133–156].

**Fig. 3:**
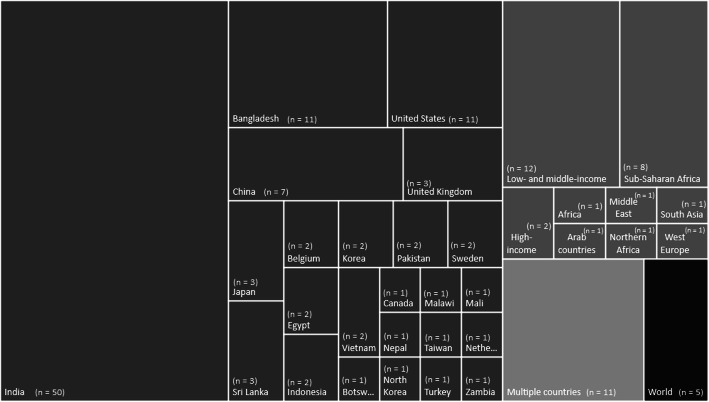
Place under study

One hundred and eleven studies used data from single countries, mostly from India (*n* = 50, 32%), followed by Bangladesh (*n* = 11, 7%, and all of them carried out in the Matlab region) and China (*n* = 7, 5%). Eleven explored data from more than one country and other 27 used data from a set of countries grouped according to income level classification or geographic region, mostly low- and middle-income countries (LMICs, *n* = 12) and sub-Saharan Africa (*n* = 9). The other five studies used global data. In Fig. 3 the area of the rectangles is proportional to the number of studies from each country or group of countries.

Estimates of mortality were derived from several sources, including civil registration systems and other official demographic statistics (29%), censuses (18%), and surveys (47%). Registration data from the Human Mortality Database, the United Nations Demographic Yearbook, and other sources of life tables and national estimates from international agencies have also been used (8%). The remaining relied on published estimates from other authors, specific studies, and experiments (16%).

Information on child age allows more than one group to be attributed to each study. Most of the publications addressed IMR, as this was the primary measure of child survival in the past. More recently, because gender discrimination seems to be more evident in the age range of 1–4 years, the focus was moved to CMR and all under-five children. In some studies, the authors defined specific age groups for the analysis, reporting on non-standard age groups [5, 7, 24, 36, 37, 39, 46, 54, 72–74, 78, 80, 81, 83, 85, 86, 88–90, 94, 99, 101, 108, 110, 111, 114, 115, 117, 118, 126, 128, 139, 146, 149, 156].

Some authors reported on female-to-male sex ratios for mortality, but most presented results as male-to-female ratios, either as a simple ratio or multiplied by 100, with values of 1 and 100, respectively, indicating equality between boys and girls. Also, in some of them, the excess mortality of girls was estimated [2, 14, 18–20, 42, 93, 102, 109, 132, 153, 154].

### Methodological characteristics

#### Reference studies

Fourteen publications defined expected values for sex ratios or sex-specific mortality based on the experience of particular populations, being categorized as *reference* studies. This heterogeneous set of studies relied upon a variety of data sources and analytical methods to define the expected differentials in male and female deaths. The first study was published in the late 1980s [77], five dated from the early 1990s [12, 93, 95, 100, 105], and the other eight from the 2010s [2, 18–20, 42, 136, 138, 153]. A summary of the characteristics is presented in Table 1. For consistency, we recalculated the outcomes and present the actual ratios of male-to-female death rates, even when the authors’ presentations were in other formats.

**Table 1:** Summary of characteristics of *reference studies*

Author, year	Observed data	Measure	Age group	Definition of expected values			Comparison method	Type of reference	Citations
				Reference population	Assessment method	Expected value			
Clark, 1987 [77]	India, published estimates	Sex ratio^a^	1–59 months	25 ‘Third World’ countries, World Fertility Surveys	Average sex ratio	99.6	Magnitude of observed and expected sex ratios, significance assessment	Descriptive	1
Johansson and Nygren, 1991 [93]	China, 1988 Two-per-thousand Fertility Survey	Sex ratio	IMR	Countries with information for at least four years 1976–1984, United Nations Demographic Yearbook	Average sex ratio	130	Magnitude of observed and expected sex ratios	Prescriptive	155
Svedberg, 1991 [95]	23 Sub-Saharan African countries, published estimates, 1953–1983	Sex ratio, Excess mortality	IMR	Sweden official statistics, 1983–1987	Sex ratio of the Swedish population		Ratio of ratios (observed/ expected) above or below the unity	Descriptive	89
			CMR						
Goodkind, 1995 [100]	Vietnam	Sex ratio	IMR	East Asian populations	Average sex ratio	97	Magnitude of observed and expected sex ratios	Descriptive	35
			CMR			95			
Hill and Upchurch, 1995 [12]	35 LMICs, DHS, 1986–1993	Sex ratio^a^	IMR	England and Wales, France, Netherlands, New Zealand, and Sweden, Coale and Demeny (1983) West model, 1820–1964	LOWESS curve for the association between sex ratios and male U5MR (from 25 to 300)	130–118	Difference between observed and expected sex ratio for each value of male mortality	Prescriptive	128
			CMR			122–103			
			U5MR			129–111			
Klasen, 1996 [105]	20 LMICs, Census and World Fertility Surveys	Sex ratio; Male mortality	IMR	Coale and Demeny (1983) West and North models and Sweden official statistics, 1983–1987	Sex ratio of reference populations		Ratio of ratios (observed/ expected) above or below the unity	Descriptive	42
			CMR						
Chaudhuri, 2011 [18]	India, 2005–06 National Family and Health Survey	Female mortality	IMR	Kerala state, India (same survey)	Linear regression for sex ratio based on male U5MR and being from Kerala	Expected female mortality derived from expected sex ratio based on the regression coefficients	Excess female mortality if observed value greater than expected; probit model	Prescriptive	NA
Srinivasan and Bedi, 2011 [42]	Tamil Nadu, India, Vital Events Survey, 1996–1999	Female mortality	IMR	Values from Waldron (1983), Johansson and Nygren (1991), Hill and Upchurch (1995), United States and United Kingdom life tables	Female mortality in function of male mortality based on the values reported in previous studies	Expected female mortality = 80% of male mortality	Difference between observed and expected values	Prescriptive	CrossRef citations: 9
Chaudhuri, 2012 [19]	13 Indian states, National Family Health Surveys, 1992, 1998 and 2005	Female mortality	IMR	Kerala state, India (same surveys)	Multivariate logistic regression	Regression coefficients	Incidence of excess female mortality = difference between observed female IMR in each state and the benchmarking female IMR (Kerala)	Prescriptive	1
Monden and Smits, 2013 [138]	35 Sub-Saharan African and Southern Asian countries, DHS, 2000s	Sex ratio	CMR	Austria, Belgium, United Kingdom, France, Germany, Netherlands, Scandinavian countries, USA, Canada, New Zealand, and Australia, Human Mortality Database, since 1920	Average sex ratio	117	Magnitude of observed and expected sex ratios	Prescriptive	14
			U5MR			125			
Jamison et al., 2013 [136]	India and China, published estimates	Sex ratio	U5MR	Demographic and health surveys from LMICs	Average sex ratio	118	Magnitude of observed and expected sex ratios; estimates of excess of female mortality based on male mortality	Descriptive	605
Alkema et al., 2014 [2]	195 countries, areas, and territories, multiple sources, 990–2012	Sex ratio	IMR	195 countries, areas, and territories, multiple sources, 1990–2012	Global relation between sex ratios and mortality levels; Bayesian model	120, 126 and 115 for IMR of 5, 20 and 150	Assessment of outlying values	Descriptive	45
			CMR			121–101 for CMR of 5 to > 30			
			U5MR			125–109 for U5MR of 20–400			
Chaudhuri, 2015 [20]	14 Indian states, National Family Health Surveys, 1992, 1998 and 2005	Female mortality	IMR	Bihar vs 13 Indian states; Bihar vs 8 less gender bias Indian states (same surveys)	Multivariate logistic regression	Coefficient of interaction between being from Bihar and female sex		Descriptive	1
Guilmoto et al., 2018 [153]	India, 2011 Census	Female mortality	U5MR	46 countries without known gender discrimination, World Population Prospects	Quadratic regression for the relation between female and male U5MRs	Expected female U5MR for each level of male U5MR	Difference between observed and expected female U5MR; absolute excess female mortality	Prescriptive	19

^a^ Sex ratio originally presented as female-to-male

*IMR* infant mortality rate; *CMR* child mortality rate; *U5MR* under-five mortality rate; *DHS* Demographic and Health Surveys; *LMICs* low- and middle-income countries; *LOWESS* Locally weighted least squares smoothing; *SD* standard deviation

Regarding the age groups under assessment, six studies reported on more than one category, usually IMR, CMR, and U5MR [2, 12, 95, 100, 105, 138], five studies only on IMR [18–20, 42, 93], and three solely on U5MR [77, 136, 153].

Several reference populations were used as the standard against which authors assessed results from one or more countries. The reference populations include: life table models from multiple high-income geographies [12, 95, 105, 138]; survey data from LMICs [18, 77, 136], official statistics gathered by the United Nations [93, 153], estimates published by other authors [42], and multiple sources including census, surveys, vital registration and surveillance systems [2]. Three studies from India used internal comparison parameters, as the authors chose the Kerala state or a set of countries as standards of low gender bias [18–20]. The studies were labeled as *prescriptive* or *descriptive*, depending on the judgment of presence or absence of gender bias in the reference populations and this information is presented in Table 1.

In five studies, after the reference population was selected, the average observed sex ratios were calculated and the resulting means were considered as the expected value for a given age group, regardless of the overall mortality level [77, 93, 100, 136, 138]. For example, Johansson and Nygren (1991) proposed an IMR sex ratio of 130 and Jamison et al. (2013) defined a reference of 118 for U5MR sex ratio [93, 136]. Another two studies proposed a range of expected sex ratio values based on overall or male mortality levels [2, 12]. For example, Hill and Upchurch [12] suggested that for male mortality rates of 25 and 300 per thousand the expected IMR sex ratio should be, respectively 130 and 118 [12]. Lastly, five works presented equations for calculating expected values, usually for female death rates according to levels of male mortality [18, 42, 153] or for the estimation of sex ratios greater than expected [95, 105]. Two studies relied upon regression coefficients for comparison [19, 20].

While the simpler studies were restricted to calculating average sex ratios as mentioned above, more sophisticated approaches included the use of locally weighted least squares (LOWESS) procedure for the association between sex ratios and male mortality [12], linear, logistic, and quadratic regression models [18–20, 153], a ratio of observed and expected sex ratios [105], and Bayesian models for the association of sex ratios and overall mortality [2].

Most studies compared the reference with observed sex ratios in single Asian countries: seven in India [18–20, 42, 77, 136, 153], two in China [93, 136], and one in Vietnam [100]. The remaining studies used data from multiple countries from Sub-Saharan Africa [105, 138], South Asia [138], low- and middle-income countries [12]. Lastly, a global study used data from 195 countries, areas, and territories with available information [2].

To evaluate whether an observed value differed from the reference population, the most frequent procedure was the simple comparison of the magnitudes of the estimates, with only a few studies reporting confidence intervals or other forms of statistical testing [2, 77, 93, 138].

#### Comparative studies

The group of 21 *comparative* studies includes works comparing sex ratios in specific populations to reference values without proposing new methodologies for the definition of expected values. The reference values are either those reflecting the experience of populations in which gender discrimination is supposed to be absent or those from areas, single country, or set of countries for comparison, with no judgment about expected values. Detailed information for each study is presented in Table 2.

**Table 2:** Summary of characteristics of *comparative studies*

Author, year	Observed data	Measure	Age group	Definition of comparison parameters		
				Reference population	Reference value (when applicable)	Comparison method
Hammoud, 1977 [68]	Algeria, Democratic Yemen, Egypt, Iraq, Jordan, Kuwait, Libyan Arab Jamahiriya, Morocco, Syrian Arab Republic and Tunisia, multiple sources, 1951–1974	Sex ratio	IMR	Mauritius, Canada, Chile, Mexico, Paraguay, United States, Hong Kong, Japan, Philippines, Thailand, Denmark, Hungary, Portugal, Yugoslavia, and Australia (United Nations and World Health Organization)	–	Magnitude of observed and expected sex ratios
			CMR			
Khosla, 1980 [69]	17 states, India, Health statistics, 1971–1975	Sex ratio	NMR	46 countries from 1970 to 1974 (WHO annual statistics)	–	Magnitude of observed and expected sex ratios
			PNMR			
			IMR			
Choe, 1987 [76]	Korea, 1974 National Fertility Survey, 1960–1974	Sex ratio	IMR	Coale and Demeny (1983) West and North models, levels 19, 20 and 21 and life tables for 10 countries (Israel, Jordan, Kuwait, Hong Kong, Sarawak, Panama, Belize, Jamaica, Guyana, Portugal)	122–133; 81–140	Magnitude of observed and expected sex ratios; Hazard models for multivariate analysis
			CMR		111–124; 71–118	
Das Gupta, 1987 [78]	Rural Punjab, India, Khanna Study, 1984	Sex ratio	U5MR^a^	Khanna 1957–1959 and Matlab Thana 1974–1977	–	Magnitude of sex ratios
Karkal, 1987 [79]	India, Sample Registration System, 1970–1980	Sex difference	0, 1, 5 years	South Asia region	–	Magnitude of sex-specific mortality and sex differences
Makinson, 1987 [80]	Egypt, 1980 World Fertility Survey	Female mortality	U5MR^a^	Coale and Demeny (1966) West model, level 13.7	Observed female mortality	Magnitude of sex-specific mortality; multivariable logistic model
Chowdhury et al., 1990 [88]	Bangladesh, Matlab Demographic Surveillance System, 1977–1985	Female mortality	NMR	Comparison of 204 sex discordant twin pairs with a random sample of 2371 singletons	Odds ratio 98	Logistic regression and McNemar’s test to assess sex differences and conditional survivorship
			IMR		Odds ratio 140	
Pebley and Amin, 1991 [39]	26 rural villages in India, Narangwal Study	Sex ratio	Under-3^a^	Study comparison area	–	Expected mortality rates 1971–1973 without intervention (control villages)
Tabutin, 1992 [97]	Algeria, Morocco, Tunisia and Egypt, multiple sources, 1965–1988	Sex ratio	IMR	United Nations model life tables for developing countries	“general pattern” for the reference countries	Magnitude of observed and expected sex ratios
			CMR			
Choe et al., 1995 [99]	China, 1988 Two-per-Thousand Survey of Fertility and Birth Control, 1965–1987	Sex ratio	IMR	Coale and Demeny (1983) West and North models, level 20, and Japan 1953–1960	119, 123, 129	Magnitude of sex ratios; multivariate proportional hazard models
			CMR		111, 113, 115	
Clark, 1995 [53]	Gwembe District, Zambia, Gwembe Study, 1956–1992	Sex-specific mortality	IMR	Twin pairs and singletons	–	Comparison of sex-specific mortality rates
Johansson, 1996 [104]	Meiji, Japan, Published estimates, 1908	Sex ratio	IMR	Swedish estimates (1750–1900), Preston standard (1976)	–	Magnitude of observed and expected sex ratios
			CMR			
Muhuri and Menken, 1997 [108]	Matlab, Bangladesh	Sex ratio	1–5 years	Study comparison area	–	Magnitude of sex ratios; logistic regression
Goodkind, 1999 [113]	North Korea, 1993 Census	Sex ratio	IMR	Previous studies (Makinson 1994; UN 1998); South Korea, China, and Taiwan	115–140	Magnitude of observed and expected sex ratios
			CMR		100–120	
Datta and Bairagi, 2000 [21]	Bangladesh, Matlab Demographic Surveillance System, 1977–1995	Sex ratio	IMR	Coale and Demeny (1983) West model and study comparison area	Excess female mortality from the equation [(observed sex ratio) - (expected sex ratio)] / (observed sex ratio)] (× 100)	
Yount, 2001 [47]	14 Middle Eastern countries, United Nations, 1970s and 1980s	Sex ratio	IMR	Same datasets for expected and observed estimates	From Hill and Upchurch (1995) estimated from the same dataset	Magnitude of observed and expected sex ratios
			CMR			
			U5MR			
Li et al., 2004 [124]	Chinese county in Shaanxi province, 1997 Household survey and community survey	Sex ratio	IMR	Published estimates of sex ratios from Li and Feldman (1996); Coale and Demeny (1983) West model	120–140	Magnitude of sex ratios; likelihood ratio test; t test; multivariate logistic regression and Cox survival
			CMR		100–120	
			U5MR		> 100	
Fuse and Crenshaw, 2006 [127]	93 countries, United Nations Statistics Division, 2000	Sex ratio	IMR	Published estimates Johansson and Nygren (1991); Hill and Upchurch (1995); Tabutin and Willems (1995)	115 to 130	Magnitude of sex ratios
Jayaraj, 2009 [132]	India, Vital Registration System (1991 and 2001) and published estimates	Female mortality	U5MR	Coale and Demeny (1966) West model, levels 18 and 19	Relative survival advantage of females (RSASF)	Magnitude of observed and expected RSAF
Oster, 2009 [36]	India, National Family Health Surveys, 1992 and 1998	Female mortality	Under-10^a^	Ethiopia, Kenya, Malawi, Namibia, Tanzania, and Zambia, DHS, 1992–2001	Regression coefficients, allowing for the interaction between being from India and female sex	Difference-in-differences
Costa et al., 2017 [14]	60 LMICs, DHS, 2005–2014	Female mortality	U5MR	Same DHS datasets for expected and observed estimates	From Hill and Upchurch (1995) and Alkema et al. (2014) estimated from the same dataset	Excess female mortality (%) = observed/ expected

^a^Specific subgroups of age (refer to Supplementary Table S2)

*IMR* infant mortality rate; *CMR* child mortality rate; *U5MR* under-five mortality rate; *DHS* Demographic and Health Surveys; *LMICs* low- and middle-income countries

The works were published since 1977, mostly from 2000 onwards. The publications reported on sets of countries (Middle East region [47], Arab countries [68], north Africa [97], sub-Saharan Africa [95], low- and middle-income countries [14], or global analyses [127]). The remaining covered one specific country (India [36, 39, 69, 78, 79, 132], China [99, 124], Bangladesh [21, 88, 108], Korea [76], Japan [104], North Korea [113], and Egypt [80]), all from Asia.

The most frequently used references were the Coale and Demeny (1966 and 1983) life tables, which are widely used for estimation and projections [157, 158]. These authors examined a large number of life tables from countries with reliable data, mostly in Europe, and used regression methods to build four families of model life tables, labeled as “West”, “North”, “East” and “South”, corresponding roughly to European regions. The models are based on the observed relationships between mortality in different age ranges and mortality levels. Each model has variants for males and females, which allows the definition of expected levels of mortality by sex or sex ratios, and which may thus be compared to values derived from a given study. Assessments of gender bias were made by comparing observed sex ratios or actual sex-specific mortality levels with expected values according to the model life tables [21, 76, 80, 99, 124, 132].

The Hill and Upchurch (1995) and the Alkema et al. (2014) references, described above, were also used in some studies, alone or combined with other methods [14, 47]. Both provide parameters that allow estimating expected sex ratios for a given level of male or overall mortality.

Intervention studies often compare their results with a control area [39, 78, 108]. Other parameters included mortality by sex in high-income countries such as Sweden [95, 104, 105] and Japan [99], sets of countries [36, 68, 69, 79], previously published expected values from other works [113, 124, 127], and singleton births as reference for sex-discordant pairs of twins [88].

#### Narrative studies

The remaining 119 publications were categorized as *narrative* as they assessed sex differentials in childhood mortality, but do not rely on any reference or expected value to corroborate the presence of bias. The assessed outcomes include absolute differences in male and female rates, sex ratios calculated either by dividing the observed sex-specific rates or derived from statistical models (as odds ratio, hazard ratio, relative risk.). This group includes studies that either did not perform statistical tests to compare rates by sex or, if these tests were included, did not account for the greater biological weakness of male children. Nonetheless, studies with higher mortality among girls are useful because this should not occur unless there is some degree of bias against girls. Also, in this group are the studies that used multivariable analyses, including sex as one of the predictors, or sex-specific models.

Up to the late 1970s, all publications used *narrative* approaches, mostly reporting on time series, with a historical perspective on mortality sex ratios [15, 27, 51, 54, 56, 57, 61, 63–67]. Almost all studies conducted with populations from high-income geographies, e.g. Belgium [4, 22], Canada [64], England [7, 30, 56], Japan [66, 114], The Netherlands [5], Sweden [118, 144], Taiwan [35], and United States [17, 51, 57, 65, 84, 135, 152] are in this group, as well as sets of high-income countries [15, 61, 122]. The approaches used in each study can be found in Supplementary Table S2.

## Discussion

In this review, we described 154 studies published since 1929 that employed quantitative methods to address sex differentials in early childhood mortality. The main challenge in these analyses is how to separate biologically determined sex differences from gender inequities that may affect mortality rates.

The studies included in our review reflect the evolution of knowledge over time across world regions. The early literature (up to the 1980s) is predominantly from high-income countries and aimed at the simple description of sex differentials. These studies established higher mortality among boys and explored potential biological mechanisms for such differences. Parameters derived from such early studies were then used in more recent years to identify populations where girls were more likely to die than would be expected. Both types of studies are relevant because early research was essential for interpreting recent findings.

Biological reasons for the lower mortality among girls include, for instance, the presence of two copies of the X chromosome, whereas male individuals have only one. The X chromosome carries more than a thousand genes and many of them are responsible for immune response, fetal development, and metabolic functions [9]. Additionally, higher odds of congenital abnormalities, lower five-minute Apgar score, need for assistant ventilation, respiratory distress syndrome in the male than in female children are some examples of male frailty [10].

Given the biological disadvantage of male children, usually, gender bias is suspected when the mortality of girls is higher than expected, which may be due to the cultural favoritism of male children resulting in neglect of girls. Although such culturally-driven ‘son preference’ is likely the main reason for higher-than-expected mortality of girls, infanticide may also affect sex ratios, although it seems to be restricted to a few societies [43, 52]; the frequency of infanticide, however, is extremely hard to measure. Whatever the reason, bias against girls is likely to affect the sex ratios, leading to either similar mortality rates for both sexes or higher mortality for girls than for boys [43, 159, 160].

Specifically, the complexities in the analyses include the specification of expected values for sex ratios for a given mortality level, the definition of what constitutes excess mortality of girls or boys, and the availability and quality of the data. In light of these conundrums, selected publications were grouped according to the methodological approach.

Fourteen studies (about 9%) aimed at presenting reference parameters, as well as analytical methods to define the expected relationship between male and female mortality. These publications are quite heterogeneous and rely either on the comparison of populations with similar mortality levels or on historical data from currently high-income populations. The most influential articles in this group include the standard developed by Hill and Upchurch (1995) based on the historical experience of high-income populations and the reference by Alkema et al. (2014) based on recent data from 195 countries [2, 12]. Besides, no statistical testing was performed in some studies, and any magnitude of differences between observed and expected values was considered. For these publications, the comparison with populations with different demographic characteristics arises as an additional limitation.

We have classified reference studies as either *descriptive* or *prescriptive*. A previous analysis employed two of the reference methods included in this review in a set of surveys from LMICs to compare their potential for identifying countries with evidence of gender bias in U5MR [14]. The authors found higher values of excess female mortality when using the expected values from Hill and Upchurch [12], a prescriptive approach, than when using Alkema et al. [2] estimates, a descriptive method [74].

Twenty-one publications (14%) were *comparative* studies that used either external or internal comparison parameters. The comparisons used in analyses included parameters defined by reference studies mentioned above, for example, the Hill and Upchurch standards. Other studies relied on Coale and Demeny’s life table models (1966 and 1983) [157, 158]. Even though these tables were not originally proposed for comparing mortality sex ratios – and therefore were not included in the group of reference studies summarized above – they have been widely used to define expected values. The limitation that arises in these situations is that model life tables incorporate estimates of typical sex differences in mortality for different levels of life expectancy for European populations, rather than for levels of child mortality [12]. Additionally, the estimates differ both according to the model and according to the level within each model [77]. It should be noted that present-day LMICs – the principal focus of the analyses – may have epidemiological and demographic profiles that differ from the settings upon which the models used as the reference were based. Additionally, the definition of the comparison group is often based on a priori*,* arbitrary judgment of absence of gender discrimination. Even so, these comparisons are useful for monitoring sex ratios and identifying outliers. Some studies combined values from published sex ratios in different populations to define expected values, which is questionable.

Most studies classified in the *narrative* group were restricted to describing sex ratios within a population, without comparisons to a reference or standard to interpret these results. Typically, sex ratios above or below the unity are assumed to be indicative of excess mortality for boys or girls. These studies are useful for providing an overview of the relationship between male and female mortality, especially in time series. Their main limitations include failing to account for the biological frailty of boys, lack of adjustment for overall mortality level, and absence of statistical assessment of the sex differences. Even so, more than identifying statistical differences between male and female mortality, it would be necessary to analyze if the relationship between the estimates is different from that that would be expected in a given context. Unless a study shows significantly higher mortality of girls than boys, which is never observed in the absence of gender bias, in general, these studies are not robust enough to provide evidence of discrimination.

Other characteristics of the publications identified in the review are discussed below. Different age ranges for mortality were used in the studies. It is important to highlight that neonatal mortality is heavily influenced by biological factors, being less sensitive to familial behavior. Post-neonatal mortality is more likely to be independent of preexisting medical complications and to happen at home, and more dependent on nurturing care provided by the family. Beyond the age of one year, the female mortality advantage is not marked as is the case for infants, and the effects of discrimination become more evident [1]. Nonetheless, there are many biomedical interventions, such as antibiotics or vaccines, that may prevent infant deaths, and for which access may be affected by gender bias in care-seeking behaviors [161].

In terms of the populations under study, most analyses came out of India, a country known for gender discrimination against females and where various interventions have been used to prevent what has been described as “daughter elimination” [43]. India, Bangladesh, and China were explored in almost half of all studies. In these three countries, there is vast evidence of female disadvantage in survival, regardless of the method used to assess it. Interestingly, few standalone studies were based on other South Asian or Middle Eastern countries, which multi-country studies often single out as presenting gender bias. Works that used data from high-income countries were mainly published up to 1980, all of them were included in the *narrative* group of studies. Most of these studies were focused on the magnitude of male disadvantage in survival. In the few studies reporting higher mortality among girls, this was only observed beyond the childhood period, particularly during adolescence or adulthood, being related to maternal deaths or those associated with the occupation.

Regarding data sources, civil or vital registration systems are the gold standard, as these record births and deaths on a continuous real-time basis and cover a whole defined population. However, few LMICs have such systems in place with sufficient coverage and quality. Therefore, household surveys collecting information on reproductive histories of women, including births and deaths of their children, constitute the main source of nationally representative child mortality estimates for LMICs. However, they may present some limitations. Since they are based on retrospective self-reports, there is always a chance that the number and sex of children have been misreported. Also, there may be errors in the dating of events and age heaping, the omission of events, and sampling errors. The same limitations apply to census data, which are also frequently used for mortality estimation using indirect methods. Waldron (1983) compared mortality sex ratios estimated from the United Nations life tables (based on vital registration) and from the World Fertility Surveys for 17 countries and found a weak correlation between these estimates, with girls presenting 12% higher mortality than boys in the WFS data, while this difference was only 5% in the life tables data for an overlapping 10-year period [8]. As mentioned above, a limitation of all data sources is that they do not allow a direct assessment of female infanticide, which would be very important for identifying gender discrimination.

The reasons behind son preference and daughter neglect vary from society to society. In some countries, the need to pay dowries for marrying daughters may impact in family’s economies, whereas in other cultures, son preference is attributed more to patriarchal systems and low female autonomy [162]. Also, religious roles that can only be performed by men may lead to the family’s preference for male children [163]. Cultural characteristics and societal values sometimes combine with limitations of resources, which leads families to choose internally which children will benefit [162, 163].

Yet, higher-than-expected male mortality could imply that boys experience mortality to a degree disproportionate to their biological disadvantage. Some evidence is provided by historical changes in sex ratios in high-income countries [11]. This study revealed that the decline in infectious diseases and the relative increase in perinatal causes led male-to-female ratios in infant mortality to increase, after which the improvement of obstetric and neonatal care led to a subsequent decline in the sex ratio [11].

We also noticed a lack of standardization in data aggregation levels. The use of national data, for example, may be problematic as they do not account for subnational variability in household and family characteristics, including behaviors influenced by son-biased values, for instance. Studies from India document important differences among the Northern and Southern states, with less evidence of gender bias in the latter [18–20, 153].

Studies assessing multiple countries often used data sources covering different periods or failing to study a representative sample of a given world region. Studies of time trends will be more informative than those reporting on single points in time, as sex ratios may vary as a function of historical elements such as war mortality, fertility transitions, and famines.

A related issue, addressed in some articles, is evidence that reductions in excess female mortality observed might be related to increasing pre-natal sex selection practices, supporting the hypothesis that infanticide and neglect of girls are being replaced by sex-selective abortion of female fetuses, contributing to the ‘missing women’ phenomenon.

Some authors argue that population sex ratios would be a more appropriate measure to assess gender bias given that these combine pre- and post-natal discrimination and account for sex-selective migration [82]. Population sex ratios were not covered in this review since we were interested primarily in methods for mortality analyses and assessment of the impact on liveborn children. Nonetheless, a few studies included in this review combined assessments of gender bias in mortality (the so-called ‘post-natal female deficit’) and prenatal sex selection [19, 29, 42, 43, 78].

The investigation of factors associated with sex ratios falls outside of the scope of the present work, but it is noteworthy that many studies complemented their survival analyses with the assessment of potential determinants including health care behaviors [14, 24, 112], health status [82], socioeconomic characteristics [31, 47, 84, 85, 90, 98, 111, 126], family composition [76, 99, 109, 110, 123, 148], fertility choices and differential stopping behavior [20, 107, 126, 141, 148]. Also, the societal position and value of girls and women, including education, power, autonomy, control over resources, and economic and social status, may affect their survival probabilities [83].

The discussion about sex differences in mortality goes beyond childhood and from a lifetime perspective, we point out the debate concerning differentials in life expectancy for males and females and the data sources and methodologies that have been a topic of discussion among researchers [164, 165].

The authors of the studies under review call for further investigation on the causes of unexpected sex differentials and the introduction of more systematic monitoring of sex differences in child mortality. Qualitative research is also necessary to document the unfair distribution of resources and discriminatory treatment of boys and girls. The mechanisms of gender discrimination are complex and multilayered, ranging from deliberate neglect in health-seeking behaviors to bias in resource allocation, which is difficult to assess solely through quantitative analyses. Moreover, additional research is needed to understand the national and regional differences in the economic and societal roles of girls and women, which seems to affect female survival.

Some caveats in the review process must be recognized. As scoping reviews do not assess study quality, some of the literature reviewed may be methodologically flawed. Also, the screening process and data extraction were not performed in duplicate. Nevertheless, our work fills a gap in the literature by summarizing studies carried out over a long-time span, and by contributing to the understanding of analyses on sex differentials in childhood mortality. The main strength of our review is the use of comprehensive combinations of terms for building the search strategy, which produced a list of about 17,000 studies to be screened. Through this search, we were able to identify the huge diversity of journals in distinct fields – such as Epidemiology, Demography, and Economics – that have published on this topic, documenting that gender and survival issues affect multiple aspects of global well-being. Additionally, the visual representation of the words used in the titles of the works reveals the diversity of terminologies, which has presented an extra challenge for the review screening process.

Rather than comparing results that emerged from analyses performed with multiple methods, our review focused on the discussion of methodological approaches for identifying gender bias and addressed the need for standardization of methodologies.

In summary, much effort has been made to measure, interpret, and explain sex differentials in early childhood mortality. Over time, there have been advances regarding data availability and quality, and in the sophistication of statistical methods on the relationship between male and female mortality, allowing definitions of what would be the so-called ‘normal’ and in the identification of unexpected differences. Yet, many authors still fail to account for the role of mortality levels in contributing to sex ratios. The issue of what constitutes the expected values of sex ratios for a given level of mortality is still open to debate but the use of model life tables and historical time series still have a role to play.

To overcome the limitations, future research on this topic should account for overall mortality levels, address different groups of age at death, and account for causes of deaths whenever possible. We also suggest the use of more than one reference value to allow interpretation of findings in light of different parameters, and the use of formal statistical testing that account for the variability of estimates of sex ratios.

## Conclusion

Addressing factors possibly associated with the sex differentials and the mechanisms underlying the biased estimates is also crucial and should cover distal as well as proximal factors. Time trends analyses, performed with a consistent methodology, can help understand both the sex differentials in a historical perspective and their relationship with changes in development level, improvements in health care, and advances in the gendered nature of social norms.

The results presented here underscore the need for further work on how to identify appropriate references for evaluating sex differentials on child mortality, leading to the formulation of policy interventions aimed at closing the unfair gender gap in childhood survival. Finally, we reinforce the call of Sustainable Development Goal 17.18 for disaggregation of data to identify more vulnerable groups within countries.

## Supplementary Information

**Additional file 1: Table S1.** PRISMA checklist. **Table S2.** Summary of characteristics of all 154 studies included in the review. **Figure S1.** Frequency of words used in the titles of the selected studies.

## Data Availability

Not applicable.

## Abbreviations

CMR: Child mortality rate
DHS: Demographic and Health Survey
IMR: Infant mortality rate
LMICs: Low- and middle-income countries
LOWESS: Locally weighted least squares
NMR: Neonatal mortality rate
PNMR: Post-neonatal mortality rate
SD: Standard deviation
U5MR: Under-five mortality rate
WFS: World Fertility Survey
