# Supplementary material for: A scoping review of methods for assessment of sex differentials in early childhood mortality
Source: BMC Pediatr. 2021 Jan 26;21:55. doi: 10.1186/s12887-021-02503-8 (PMC7836200; doi:10.1186/s12887-021-02503-8)
Supplement: Supplementary file 1 — Additional file 1: Table S1. PRISMA checklist. Table S2. Summary of characteristics of all 154 studies included in the review. Figure S1. Frequency of words used in the titles of the selected studies. [file 12887_2021_2503_MOESM1_ESM.docx]

Supplementary Table S1 – PRISMA checklist.

| **Section/topic** | **#** | **Checklist item** | **Reported on page #** |
| --- | --- | --- | --- |
| **TITLE** | | |  |
| Title | 1 | Identify the report as a systematic review, meta-analysis, or both. | 1 |
| **ABSTRACT** | | |  |
| Structured summary | 2 | Provide a structured summary including, as applicable: background; objectives; data sources; study eligibility criteria, participants, and interventions; study appraisal and synthesis methods; results; limitations; conclusions and implications of key findings; systematic review registration number. | 2 |
| **INTRODUCTION** | | |  |
| Rationale | 3 | Describe the rationale for the review in the context of what is already known. | 3 |
| Objectives | 4 | Provide an explicit statement of questions being addressed with reference to participants, interventions, comparisons, outcomes, and study design (PICOS). | NA |
| **METHODS** | | |  |
| Protocol and registration | 5 | Indicate if a review protocol exists, if and where it can be accessed (e.g., Web address), and, if available, provide registration information including registration number. | NA |
| Eligibility criteria | 6 | Specify study characteristics (e.g., PICOS, length of follow-up) and report characteristics (e.g., years considered, language, publication status) used as criteria for eligibility, giving rationale. | 4 |
| Information sources | 7 | Describe all information sources (e.g., databases with dates of coverage, contact with study authors to identify additional studies) in the search and date last searched. | 4 |
| Search | 8 | Present full electronic search strategy for at least one database, including any limits used, such that it could be repeated. | Fig. 1 |
| Study selection | 9 | State the process for selecting studies (i.e., screening, eligibility, included in systematic review, and, if applicable, included in the meta-analysis). | 4 |
| Data collection process | 10 | Describe method of data extraction from reports (e.g., piloted forms, independently, in duplicate) and any processes for obtaining and confirming data from investigators. | 4 |
| Data items | 11 | List and define all variables for which data were sought (e.g., PICOS, funding sources) and any assumptions and simplifications made. | 4 |
| Risk of bias in individual studies | 12 | Describe methods used for assessing risk of bias of individual studies (including specification of whether this was done at the study or outcome level), and how this information is to be used in any data synthesis. | NA |
| Summary measures | 13 | State the principal summary measures (e.g., risk ratio, difference in means). | NA |
| Synthesis of results | 14 | Describe the methods of handling data and combining results of studies, if done, including measures of consistency (e.g., I^2^) for each meta-analysis. | NA |

NA = Not applicable

Supplementary Table S2 – Summary of characteristics of all 154 studies included in the review.

| Study identification | Place | Mortality data source | Period of data | Age group | Analysis type | Measure | Reference population | Comparison method |
| --- | --- | --- | --- | --- | --- | --- | --- | --- |
| Abernethy, V and Yip, R (1990) | Tennessee, United States | Birth-data records | 1976-1983 | Post-neonatal | Narrative | M:F ratio | NA | Analysis of variance |
| Abramowicz, M and Barnett, HL (1970) | New York, United States and 13 countries (Australia, Chile, Denmark, England and Wales, Guatemala, Jamaica, Japan, Luxembourg, Mexico, New Zealand, Sweden, Switzerland, and United States) | NY: Department of Statistics, New York city Department of Health  Countries: United Nations Publication, 1954 | NY 1870-1960, countries 1915-1949 | Infant | Narrative | M:F ratio | NA | Scatter plot |
| Agnihotri, SB (2001) | India | Sample Registration Survey | 1982-1997 | Infant Child | Narrative | M-F difference F-M difference slope of linear regression | NA | Linear regression |
| Ahmed, FAE (1990) | Egypt | Census | 1976 | Infant Under-two Under-three Under-five | Narrative | M:F ratio | NA | Magnitude of sex ratios; OLS regression |
| Alam N, et al (2007) | Matlab, Bangladesh | Demographic Surveillance System | 1976-1999 | Neonatal Post-neonatal Child | Narrative | M:F odds ratio | NA | Sex-specific mortality over time; descriptive sex ratio by birth cohort; logistic regression by cohort and area |
| Alam, N and Bairagi, R (1997) | Matlab, Bangladesh | Demographic Surveillance System | 1978-1994 | Child | Narrative | M:F ratio F:M ratio | NA | Sex-specific mortality and sex ratio over time; logistic regression for each birth cohort and comparison to control area |
| Alkema, L et al (2014) | 195 countries, areas and territories | Survey data, censuses, vital registration systems, sample registration and surveillance systems | 1990-2012 | Infant Child Under-five | Reference, descriptive | M:F ratio; Excess female mortality | Same 195 countries with information (relationship between own data) | Bayesian hierarchical models for identifying outliers |
| Alter, G et al (2004) | five sets of Eurasia Project villages (China, Japan, Italy, Belgium, Sweden) | Eurasia Project | 1789-1899 | Infant Child | Narrative | M:F ratio | NA | Magnitude of sex ratios |
| Altindag, O (2016) | Turkey | Demographic and Health Survey | 1993, 1998, 2003, and 2008 | Infant | Narrative | F-M difference | NA | Difference-in-differences regression model |
| Amin, S (1990) | 26 villages in the district of Ludhiana, rural Punjab, India | Narangwal Study | 1964-1973 | Neonatal (0-1 month) Postneonatal (2-12 months) Childhood (13-36 months) Under-three | Narrative | Relative risk | NA | Multivariate hazard models |
| Arnold, F (1992) | 26 low- and middle-income countries | Demographic and Health Survey | 1986-1989 (mortality in the 10 years preceding the interview) | Neonatal Post-neonatal Infant Child | Narrative | M:F ratio | NA | Sex ratio above or below one |
| Arnold, F (1997) | 44 low- and middle-income countries | Demographic and Health Survey | 1986-1995 (mortality in the 10 years preceding the interview) | Neonatal Post-neonatal Infant Child Under-five | Narrative | M:F ratio | NA | Magnitude of sex ratios; significance assessment |
| Arnold, F et al (1998) | India | National Family Health Survey | 1992-1993 | Child | Narrative | Excess female mortality (%); Relative Risk | NA | Multivariate hazard models |
| Arokiasamy, P (2004) | India | National Family Health Survey | 1992-1993 (births in the four years preceding the interview) | Neonatal Post-neonatal Child | Narrative | F:M ratio; Odds ratio | NA | Logistic regression |
| Arokiasamy, P (2007) | India | National Family and Health Survey | 1992 and 1998 (mortality in the 10 years preceding the interview) | Neonatal Post-neonatal Child | Narrative | F:M ratio | NA | % excess based on sex ratio; multivariate logistic regression model |
| Ashorn, P et al (2002) | Lungwena, rural Malawi | Prospective cohort | NA | Under-three | Narrative | M:F relative risk | NA | Cox regression |
| Attané, I (2009) | Chinese provinces, autonomous regions, and municipalities | Census | 2000 | Infant | Narrative | F:M ratio | NA | Correlation and multiple linear regression |
| Bakwin, H (1929) | United States | United States Birth, Stillbirth, and Infant Mortality Statistics | 1918-1924 | Infant | Narrative | M:F ratio | NA | Sex ratio above or below one |
| Balsara, SL et al (2013) | United States | NCHS and CDC linked birth and deaths records | 1999-2007 | Infant Child | Narrative | M:F relative risk; M-F difference of rates | NA | Magnitude of relative risks and difference in number of deaths |
| Basu, AM (1989) | Uttar Pradesh and Tamil Nadu, India | Survey of cultural influences on demographic behavior | NA | Under-five | Narrative | Sex-specific mortality; M:F ratio | NA | Magnitude of sex-specific mortality and sex ratios |
| Berman, SM et al (1987) | Georgia, United States | Linked birth certificate-death certificate records | 1974-1981 | Neonatal | Narrative | M:F ratio | NA | Mantel-Haenszel test |
| Bhargava, A (2003) | Uttar Pradesh, India | National Family Health Survey | 1992-1993 (births in 1982-1992) | Infant | Narrative | Maximum likelihood estimates | NA | Probit models |
| Bhaumik, U et al (2004) | Massachusetts, Unites States | Linked birth and death certificate data | 1989-1995 | Neonatal Post-neonatal Infant | Narrative | F-M difference | NA | Chi-squared test; Non-linear regression |
| Bhuiya, A and Streatfield, K (1991) | Matlab, Bangladesh | Demographic Surveillance System | births in 1982 | 0 month 1-5 months 6-11 months 12-17 months 18-35 months | Narrative | Risk of dying by sex Hazard logit coefficients by sex | NA | Multivariate hazard model; maximum-likelihood logit model |
| Boco, AG (2014) | 30 sub-Saharan African countries | Demographic and Health Survey | 2004-2012 (births in the five years preceding the interview) | Under-five | Narrative | M:F odds ratio | NA | Multilevel discrete-time hazard models |
| Bourne, KL and Walker, GM (1991) | The 14 major states and Delhi, India | Census | 1981 | Under-one Under-two Under-three Under-five | Narrative | Actual rates and median | NA | Sign test (F> M or M>F), median polish |
| Caldwell, P and Caldwell, JC (1990) | India | Sample Registration System, Census | 1976-1981 | Under-five | Narrative | F:M ratio | NA | Magnitude of sex ratios |
| Chaudhuri, S (2011) | India | National Family Health Survey | 2005-2006 | Infant | Reference, prescriptive | Excess female mortality | Kerala state, India (same dataset) | Linear regression for sex ratio based on male U5MR and being from Kerala; difference between observed and expected female mortality; probit model for the likelihood of excess female mortality |
| Chaudhuri, S (2012) | 13 states, India | National Family Health Survey | 1992-1993, 1998-1999, and 2005-2006 (births in 1950-2010) | Infant | Reference, prescriptive | Incidence of missing girls | Kerala state, India (same dataset) | Multivariate logistic regression |
| Chaudhuri, S (2015) | Bihar | National Family Health Survey | 1992, 1998, and 2005 | Infant | Reference, prescriptive | Excess female mortality (%) Odds Ratio | 13 states and India as whole, 8 states (same dataset) | Multivariate logistic regression; proportion of excess female deaths; Hill and Upchurch (1995) estimates |
| Chen, J et al (2007) | rural areas of China | National Family Planning and Reproductive Health Survey | 2001 (births between 1989-2000) | Infant | Narrative | Odds ratio | NA | Multilevel logistic regression models with Markov Chain Monte Carlo method |
| Choe, M et al (1998) | Egypt, Bangladesh, and Republic of Korea | Egyptian Demographic and Health Survey; Bangladesh Fertility Survey; Korean Family and Health Survey | 1988, 1989, 1991 | 2-5 years Under-five | Narrative | M:F ratio, odds ratio, relative risk | NA | Discrete time hazard model, logistic regression |
| Choe, MK (1987) | Korea | World Fertility Survey | 1974 (births in 1960-1965 and 1966-1974) | Infant Child | Comparative, not standard | M:F ratio | West and North life tables (Coale and Demeny, 1983) and life tables for 10 countries (Israel, Jordan, Kuwait, Hong Kong, Sarawak, Panama, Belize, Jamaica, Guyana, Portugal) | Magnitude of sex ratios; multivariate hazard models by sex |
| Choe, MK et al (1995) | China | Two-per-Thousand Survey of Fertility and Birth Control | 1988 (births between 1965-1987) | Infant Child (1-5 years) | Comparative, standard | M:F ratio | West and North life tables, level 20 (Coale and Demeny, 1983) and Japan (1953-1960) | Magnitude of sex ratios; Hazard models for firstborn and later-born children |
| Chowdhury, MK et al (1990) | Matlab, Bangladesh | Demographic Surveillance System | 1977-1985 | Neonatal Infant Under-nine | Comparative | F:M odds ratio, conditional survivorship | Singletons from the same population | Survival analysis by sex; McNemar's test |
| Chowdhury, R (2017) | Haryana, India | Cluster randomized trial examining the Integrated Management of Neonatal and Childhood Illness programme | 2008-2010 | 1st day 1st week  Neonatal Post-neonatal | Narrative | Odds ratio | NA | Multiple logistic regression |
| Chunkath, SR and Athreya, VB (1997) | Tamil Nadu, India | Directorate of Public Health Survey | 1995 | Post-neonatal Infant Early, late neonatal | Narrative | F-M difference F:M ratio | NA | Differences and ratios by district |
| Clark, A (1987) | India | Published estimates | 1970-1978 | Post-neonatal under-five | Reference, descriptive | F:M ratio | World Fertility Surveys from 25 countries | Average sex ratio and significance assessment based on confidence limits |
| Clark, S et al (1995) | Gwembe District, Zambia | Gwembe study (longitudinal) | 1956 and 1992 | Infant | Comparative, not standard | sex-specific mortality rates | twin pairs and singletons | Magnitude of sex-specific mortality rates |
| Cleland, J and Harris, K (1998) | 27 low- and middle-income countries | Demographic and Health Survey | DHS - phase I (births in the 10 years preceding to the interview) | 6-60 months | Narrative | M:F ratio | NA | Sex ratio above or below one, Cox-Mantel test |
| Costa, JC et al (2017) | 60 low- and middle-income countries | Demographic and Health Survey | 2005-2014 | Under-five | Comparative, standard | Excess female mortality (observed/expected) | Demographic and Health surveys (same datasets for expected and observed estimates) using expected values from Hill and Upchurch (1995) and Alkema et al (2014) | Excess female mortality (%) |
| Crognier, E et al (2006) | southern Morocco and Bolivian Altiplano | Health and Fertility Survey of the Province of Marrakech, Survey by the Bolivian Institute of Altitude | 1984 and 1998-1999 | 0-2 years 2-5 years | Narrative | Percentage of surviving offspring by sex | NA | Survival analysis employing Kaplan-Meier estimator |
| Das Gupta, M (1987) | rural Punjab, India | Khanna study (survey) | 1984 | Neonatal Post-neonatal Infant 12-23 months 24-59 months 0-59 months | Comparative, not standard | M:F ratio | Khanna 1957-1959 and Matlab Thana 1974-1977 | Magnitude of sex ratios |
| Das Gupta, M and Bhat, PNM (1997) | India | Census | 1981 and 1991 | Under-five | Narrative | M:F sex ratio | NA | Percentual of excess female mortality based on sex ratio |
| Datta, A and Bairagi, R (2000) | Matlab, Bangladesh | Demographic Surveillance System | 1978-1995 | Infant | Comparative, standard | F:M sex ratio, excess female mortality | West model life table (Coale and Demeny, 1983) and study comparison area | Magnitude of sex ratios and estimates of excess female mortality |
| Devos, I (1996) | Belgium | Published estimates | 1890-1910 | Infant Child | Narrative | M:F ratio | NA | Sex ratio above or below one |
| Diamond-Smith, N et al (2020) | 153 districts, India | Census | 2000 and 2011 | 0-6 years | Narrative | F:M ratio | NA | Change in sex ratio over time |
| Drevenstedt, GL et al (2008) | 15 high-income countries | Human Mortality Database | 1751-2004 | Infant | Narrative | M:F ratio | NA | Linear regression of sex ratio on overall mortality level |
| D'Souza, S and Chen, LC (1980) | Matlab, Bangladesh | Demographic Surveillance System | 1974-1977 | Neonatal Post-neonatal Infant Child | Narrative | Sex-specific mortality rates F:M ratio | NA | Significance assessed using t-test |
| Eggerickx, T and Tabutin, D (1994) | Belgium | Census | 1890 | Infant Child | Narrative | M:F ratio | NA | Sex ratio above or below one |
| El-Badry, MA (1969) | Ceylon, India and Pakistan | United Nations Demographic Yearbook, published estimates, Abridged life tables, Population Growth Experiment | 1953-1965 | Infant Child | Narrative | Sex-specific mortality rates F:M ratio | NA | Magnitude of sex ratios |
| Fauveau, V et al (1991) | Matlab, Bangladesh | Demographic Surveillance System | 1986-1987 | Child | Narrative | F:M relative risk | NA | Incidence density ratios |
| Finlay, R (1981) | Cartmel, Cumbria, Englnd | Cartmel family reconstitution and probate records | 1600-1750 | Infant Child | Narrative | M:F ratio | NA | Magnitude of sex-specific mortality |
| Flato, M and Kotsadan, A (2015) | 29 low- and middle-income countries | Demographic and Health Survey | 1986-2011 | Infant | Narrative | Sex-specific rate | NA | Magnitude of estimates |
| Fuse, K and Crenshaw, EM (2006) | 93 countries | United Nations | 2000 | Infant | Comparative, standard | M:F ratio | Hill and Upchurch (118 to 130), Tabutin and Willems (110), Johansson and Nygren (130) | Magnitude of sex ratios, correlation; OLS regression |
| Garenne, M (2003) | 25 sub-Saharan African countries | Demographic and Health Survey and World Fertility Survey | 1977-1998 | Neonatal Post-neonatal Child | Narrative | M:F sex ratio | NA | Student's t test; chi-square test |
| Gellatly, C and Petrie, M (2017) | India | National Family Health Survey | 1992-1993, 1998-1999, and 2005-2006 | Infant | Narrative | Mortality hazard M:F ratio | NA | Logistic regression |
| Gleason, SM (2003) | India | Census | 1981 | Under-five | Narrative | Marginal effects from the sex-specific regression models | NA | Robust “seemingly unrelated regression” method |
| Goodkin, D (1995) | Vietnam | Census | 1979 and 1989 | Infant Child | Reference, descriptive | M:F sex ratio | Variety of populations in East Asia | Magnitude of sex ratios |
| Goodkin, D (1999) | North Korea | Census | 1993 | Infant Child | Comparative, not standard | M:F ratio | Previous studies (Makinson 1994; UN 1998) expected IMR = 115-140 and CMR = 100-120; South Korea, China and Taiwan | Magnitude of sex ratios |
| Guilmoto, C et al (2015) | Indonesia | Census | 2010 | before 15-19 years | Narrative | M:F ratio | NA | Magnitude of sex ratios |
| Guilmoto, C et al (2018) | Indian states and districts | Census | 2011 | Under-five | Reference, prescriptive | Excess female mortality | 46 countries without known sex selection in mortality (World Population Prospects, 1950-2015); quadratic regression for the association between female and male mortality rates | Comparison of observed and expected female mortality rates |
| Hammoud, EI (1965) | 12 countries | United Nations Demographic Yearbook | 1954-1955 | Infant | Narrative | M:F ratio | NA | Magnitude of sex ratios; rank correlation of infant mortality rates and sex ratios |
| Hammoud, EI (1977) | Algeria, Democratic Yemen, Egypt, Iraq, Jordan, Kuwait, Libyan Arab Jamahiriya, Morocco, Syrian Arab Republic and Tunisia | United Nations Demographic Yearbook, WHO World Health Statistics Annual (1971 and 1972), national publications | 1951-1974 | Infant Child | Comparative, not standard | M:F ratio | Mauritius, Canada, Chile, Mexico, Paraguay, United States of America, Hong Kong, Japan, Philippines, Thailand, Denmark, Hungary, Portugal, Yugoslavia and Australia (United Nations and World Health Organization) | Magnitude of sex ratios |
| Harpending, HC and Pennington, R (1991) | Botswana | Family survey system | ]1959-1975[ | Infant Child | Narrative | Sex-specific mortality rates | NA | Chi-square test |
| Hill, K and Upchurch, D (1995) | 35 low- and middle-income countries | Demographic and Health Survey and World Fertility Survey | 1980-1990; 1970-1980 | Infant Child Under-five | Reference, prescriptive | F:M ratio Female disadvantage index | Five countries from 1800 to 1964 (subset of Coale and Demeny (1983) life tables); LOWESS curve for the association between sex ratios and male under-five mortality | Index of female disadvantage (observed - expected sex ratios) |
| Holmes SJ and Mentzer VP (1931) | 19 countries | Published estimates | 1911-1929 | Infant 2-3 months 4-12 months | Narrative | M:F ratio | NA | Magnitude of sex ratios |
| Humphrey, L et al (2012) | London, England | Burial and baptism registers | 1750-1839 | Neonatal Infant | Narrative | M:F relative risk Coefficients from sex-specific biometric model | NA | Significance of relative risks, biometric model by sex |
| Iqbal, N et al (2018) | 195 countries | UNICEF | 2015 | Under-five | Narrative | M:F ratio Sex-specific U5MR Excess female mortality | NA | Multivariable linear regression; likelihood ratio test |
| Jamison, DT (2013) | India and China | Demographic and Health Survey | 2000s | Under-five | Reference | M:F ratio | Demographic and Health Surveys from low- and middle-income countries | Magnitude of sex ratios and estimates of excess female mortality |
| Janssens, A et al (2010) | The Netherlands | Death registration (community level) and Population Registers (individual level) | 1875–1899 | Infant 1 year 2-4 years | Narrative | Age-specific mortality ratios by sex M:F ratio Propensity to die | NA | Description of sex ratios, discrete-time multi-level event history analysis using logistic regression |
| Jayaraj, D (2009) | India | Vital Registration System and published estimates | 1991 and 2001 | Under-five | Comparative | Relative survival advantage of females (RSAF) | RSAF for West model life tables of levels 18 and 19 (Coale and Demeny, 1966) | Comparison of RSAF magnitude |
| Johansson, S (1996) | Meiji, Japan | Published estimates | 1908 | Infant Child | Comparative, standard | M:F ratio | Swedish estimates (1750-1900), Preston standard (1976) | Magnitude of sex ratios |
| Johansson, S and Nygren, O (1991) | China | Fertility survey by State Family Planning Commission | 1988 | Infant | Reference, prescriptive | Sex ratio Excess female mortality | Developed countries and developing countries without strong sex preferences, from 1976 to 1984 (1985 UN Demographic Yearbook) | Magnitude of sex ratios |
| Kalsi, P (2017) | India | District Level Household Survey | 1987-2001 | Infant Child | Narrative | Regression coefficients (dependent binary variable for dead children) | NA | Difference-in-difference-in-differences regression model |
| Karkal, M (1987) | India | Sample Registration System | 1970-1980 | 0, 1, 5 years | Comparative, not standard | F-M difference | South Asia (1970-1975 and 1976-1980) | Magnitude of sex differences |
| Karlsson, O et al (2019) | India | National Family Health Survey | 2005-2006 and 2015-2016 | Neonatal Post-neonatal Child | Narrative | Mortality probability | NA | Sex-specific logit model |
| Khosla, T (1980) | 17 Indian states | Health Statistics of India | 1970 | Neonatal Post-neonatal Infant | Comparative, not standard | M:F ratio | 46 countries from 1970-1974 (WHO annual statistics for 1977) | Simple comparison of sex ratios magnitude |
| Kikuzawa, S (1999) | Yokouchi, Japan | Annual census registers | 1671-1871 | Under-three | Narrative | Sex-specific mortality Coefficients of sex-specific regression models | NA | Magnitude of sex-specific mortality; logistic regression |
| Kishor, S (1993) | India | Indian Development District Database (census + other sources) | 1981 | Under-five | Narrative | logged female mortality, male mortality and F:M ratio | NA | "Spatial disturbance model" approach and maximum likelihood estimation techniques |
| Klasen, S (1996) | Sub-Saharan Africa | World Fertility Surveys, Census, Urban surveys, Rural surveys | 1954-1983 | Infant Child | Reference, prescriptive | Sex ratio | Coale and Demeny (1983) West model life tables (non-European populations), North model (Northern European) and Swedish data | Excess male mortality = observed sex ratio / expected sex ratio (from reference population) |
| Koenig, MA and D'Souza, S (1986) | Matlab, Bangladesh | Demographic Surveillance System | 1966-1973 and 1973-1974 | 1-60 mo | Narrative | Cumulative probabilities F:M ratio | NA | Life-table probabilities by sex |
| Krishnan, A et al (2013) | India | Ballabgarh Health and Demographic Surveillance System | 2006-2011 | Neonatal Under-three | Narrative | F:M hazard ratios | NA | Kaplan-Meier survival curves, log rank test |
| Krishnan, A et al (2014) | India | Ballabgarh Health and Demographic Surveillance System | 1972-1997 | Neonatal Post-neonatal Child | Narrative | Sex-specific rates | NA | Chi-squared test, linear regression |
| Kumar, G (1989) | India | Official Life Tables, Registrar General of India and Mitra | 1941-1950, 1951-1960, 1961-1970 | 0, 1, 2, 3, 4 years | Narrative | M:F ratio Sex-specific rates | NA | Magnitude of estimates |
| Kuntla, S et al (2014) | India | National Family and Health Survey | 1992-2006 | Child | Narrative | F-M difference | NA | Decomposition analyses |
| Langford, C and Storey, P (1993) | Sri Lanka | Census | 1921-1981 | Infant Child | Narrative | M:F ratio | NA | Magnitude of sex ratios |
| Langford, CM (1984) | Sri Lanka | Reports of the Registrar General of Ceylon on Vital Statistics, Bulletins on Vital Statistics and census | 1921-1971 | Infant Child | Narrative | M:F ratio | NA | Sex ratio above or below one |
| Langsten, R (1981) | Matlab, Bangladesh | Vital registration system of the Matlab field research and published estimates | 1966-1977 | Infant Child | Narrative | F:M ratio | NA | Sex ratio above or below one |
| LeGrand, TK and Mbacke, CS (1995) | Bamako, Mali; Bobo-Dioulasso, Burkina Faso; Fissel and Thienaba, rural Senegal | Enquête sur la mortalité infantile dans le Sahel | births in 1981-1983 | 1-23 months | Narrative | F:M ratio | NA | Discrete hazard regression |
| Li, J and Lavely W (2003) | Diandong county (a pseudonym) | Survey | 1994 (births in the three years preceding the interview) | Infant | Narrative | Sex-specific mortality | NA | Sex-specific mortality |
| Li, S et al (2004) | China | Household survey and community survey | 1997 (deaths from 1994 to 1996) | Neonatal Postneonatal Child | Comparative | M:F ratio | Published estimates from Li and Feldman (1996) and West model life table (Coale and Demeny, 1983) | Magnitude of sex ratios; likelihood ratio test; t test; multivariate logistic regression and Cox survival |
| Lin, M-J et al (2014) | Taiwan | National Birth Registries and Death Registries | 1980-1992 for births and 1980-1996 for deaths | Neonatal (also death at 6, 12, 18, 24, 36 and 48 months) | Narrative | Sex-specific rates F:M ratio | NA | Sex-specific mortality rates by parity; regression models of deaths allowing for the interaction terms including sex |
| Maitra, P and Rammohan, A (2011) | 15 major states in the country, India | National Family Health Survey | 1998-1999 | Infant | Narrative | Sex-specific probabilities | NA | Probit model |
| Makinson, C (1987) | Egypt | World Fertility Survey | 1980 | 0 months 1-2 months 3-11 months 12-23 months 24-59 months | Comparative, standard | Sex-specific rates | West model life table, level 13.7 (Coale and Demeny, 1983) | Magnitude of sex-specific mortality, multivariable logistic regression |
| Martin, WJ (1951) | England and Wales | Official statistics | 1841-1947 | Infant Under-five | Narrative | M:F ratio | NA | Magnitude of sex ratios |
| Mbacke, CSM and LeGrand, TK (1992) | Mali | Demographic and Health Survey | 1987 (births in the five years preceding the interview) | 0, 1-2, 3-5, 6-11, 12-23, 24-59 months Neonatal Post-neonatal Child | Narrative | F:M ratio | NA | Magnitude of sex ratios |
| McMillen, MM (1979) | United States | Vital statistics | 1922-1936 and 1950-1972 | Infant | Narrative | M:F ratio | NA | Magnitude of sex ratios |
| McNay K, et al (2005) | England and the Wales | Vital Registration System | 1851-1860 | 0–9 years | Narrative | F:M ratio | NA | Sex ratio above or below one |
| Modin, B (2002) | Sweden | Uppsala Birth Cohort Study | 1915-1929 | Infant 1-10 years | Narrative | Odds ratio Relative risk | NA | Logistic regression (infancy) and Poisson (childhood) for boys and girls, separatly |
| Monden, CWA and Smits, J (2012) | 35 sub-Saharan African and southern Asian countries | Demographic and Health Survey | 2000s | Neonatal Post-neonatal Infant  Child Under-five | Reference, prescriptive | M:F ratio | Austria, Belgium, UK, France, Germany, Netherlands, Scandinavian countries, USA, Canada, New Zealand, and Australia (Human Mortality Database, 1920 up to U5MR < 2.5%) | Magnitude of sex ratios |
| Muhuri, PK and Menken, J (1997) | Matlab, Bangladesh | Matlab Census and the Demographic Surveillance System | 1982 | 1-5 years | Comparative, not standard | F:M ratio | Study comparison area | Magnitude of sex ratios; logistic regression |
| Muhuri, PK and Preston, SH (1991) | Matlab, Bangladesh | Matlab Census and the Demographic Surveillance System | 1981-1982 | 6 months - 5 years | Narrative | F:M ratio Odds ratio | NA | Magnitude of sex ratios; logistic regression |
| Murthi, M et al (1995) | 296 districts in 14 of the 15 most populous states, India | Census | 1981 | Under-five | Narrative | Female disadvantage | NA | female disadvantage = 100 * (q5f-q5m)/q5f (negative values indicate female advantage) |
| Nadarajah, T (1983) | Sri Lanka | Census | 1952-1972 | Infant Child | Narrative | M:F ratio | NA | Magnitude of sex ratios |
| Nandi, A (2015) | India | District Level Household Survey | 2002-2004 | Infant | Narrative | Sex-specific percentage of deaths | NA | Difference-in-differences regression model |
| Narayana, D (2008) | 15 Indian states, districts of Tamil Nadu | Sample Registration System, Danish International Development Agency project on healthcare | 1971-2005, 1998 | Infant | Narrative | F:M ratio, annual percentage reduction by sex | NA | Magnitude of estimates |
| Oster, E (2009) | India | National Family Health Survey | 1992 and 1998 (births in 10 years preceding the interview) | Under-ten, including <6mo, 6mo-1year, 1-2yrs, 2-4yrs, 4-6yrs, 6-8yrs, 8-10yrs | Comparative, not standard | Interaction between female sex and birth in India | DHS from sub-Sahara African countries (Ethiopia, Kenya, Malawi, Namibia, Tanzania, and Zambia) | Difference-in-differences to compare mortality in India and sub-Saharan Africa |
| Padmanabha, P (1982) | India | Sample Registration System | 1970-1978 | Infant Child | Narrative | sex-specific mortality rates F:M ratio | NA | Magnitude of sex ratios |
| Park, H (2018) | Unyang, Korea | Family register data | 1909-1977 | 1-5 years | Narrative | Sex-specific probabilities | NA | Logistic regression |
| Patra, N (2011) | India | National Family Health Survey, Sample Registration System | 1992-1993, 1998-1999 and 2005-2006 (births in the five years preceding the interview) | Infant | Narrative | F:M ratio | NA | Magnitude of sex ratios |
| Pebley, AR and Amin, S (1991) | Ludhiana district, Punjab, India | Narangwal Study | 1968-1973 | Infant 1-3 years 0-3 years | Comparative, standard | M:F ratio | Control region (with no intervention) | Magnitude of sex ratios |
| Pham, TL et al (2012) | Vietnam | Demographic and Health Survey | 1997 and 2002 (births between 1961 and 2002) | 0–1, 1–6, 6–12, 12–24, 24–60, and > 60 months | Narrative | Sex-specific no-frailty coefficients | NA | Proportional hazard model |
| Pongou, R et al (2012) | 31 sub-Saharan Africa | Demographic and Health Survey | 1986-2006 | Infant | Narrative | Sex effect in mortality | NA | Linear probability model |
| Pongou, R et al (2017) | 31 sub-Saharan Africa | Demographic and Health Survey | 1987-2005 | Infant | Narrative | M-F difference | NA | Regression-based decomposition analysis |
| Raj, A et al (2019) | India | National Family Health Survey | 1992, 1998 and 2005 (births within one to five years preceding the interview) | Infant | Narrative | Odds ratio | NA | Logistic regression |
| Ren, XS (1995) | Shaanxi, Liaoning, and Guangdong provinces, China | China In-depth Fertility Survey | 1985 and 1987 | Neonatal Post-neonatal Child | Narrative | M:F ratio log odds | NA | Logistic regression |
| Rose, E (1999) | India | Additional Rural Incomes Survey | 1969-1971; surviving children born between 1961 e 1964 | Up to school age | Narrative | Ratio of survival probabilities | NA | Multinomial logit model |
| Rosenblum, D (2012) | India | Reproductive and Child Health Survey | 2002-2004 | Under-five | Narrative | Sex-specific mortality rates | NA | Ordinary least squares regression and logit estimation |
| Rosenstock, S et al (2013) | Nepal | Newborn Washing Study (clinical trial) | 2002-2006 | Neonatal | Narrative | Odds ratio | NA | Chi-square test, t test and binomial regression model |
| Rosenzweig, MR and Schultz, TP (1982) | India | Household sample, Census | 1971, 1961 | Under-five | Narrative | M-F difference | NA | OLS regression |
| Rutstein, SO (1984) | 41 low- and middle-income countries | World Fertility Survey | births in the 10 years preceding the survey | Neonatal Post-neonatal Infant Under-five Toddler (1 year) Child (2-3 years) | Narrative | M:F ratio | NA | Magnitude of sex ratios |
| Sabir, NI and Ebrahim, GJ (1984) | Lahore, Pakistan | Household survey | NA | Infant 1-2 years >2 years | Narrative | Number and percent of deaths by sex | NA | Number and percent of deaths by sex |
| Sathar, ZA (1987) | Pakistan | Census, Population Labour Force and Migration Survey, and Micro-nutrient Survey | 1965-1979 | Neonatal Post-neonatal Child 1-2 years | Narrative | Sex-specific mortality rates M:F ratio | NA | Magnitude of sex ratios |
| Sawyer, CC (2012) | World | Survey data, censuses, vital registration systems, sample registration and surveillance systems | 1970s, 1980s, 1990s, and 2000s | Infant Child Under-five | Narrative | M:F ratio | NA | method from UN IGME |
| Shapiro, S (1954) | United States | Birth certificates | 1951 | Neonatal | Narrative | sex-specific mortality rates | NA | Magnitude of sex-specific mortality rates |
| Silverman, JG et al (2011) | India | Demographic and Health Survey | 2005-2006 | Infant Under-five | Narrative | Relative risk | NA | Cox proportional hazard models |
| Srinivasan, S and Bedi, AS (2008) | Tamil Nadu, India | Vital Events Surveys | 1996-1999 and 2003 | Infant | Narrative | Sex-specific number of deaths and mortality rates F-M difference | NA | Magnitude and significance of sex ratios |
| Srinivasan, S and Bedi, AS (2011) | Tamil Nadu, India | Vital Events Surveys | 1996, 1997, 1998, 1999 and 2003 | Infant | Reference, descriptive | Female mortality | Published estimates (Waldron, 1983; Johansson and Nygren, 1991; Hill and Upchurch, 1995; life tables from United States and United Kingdom) to define the expected female mortality based on male mortality (expected to be about 77-85% of male mortality) | Expected female = 0.8 male mortality; deficit = observed - expected |
| Steen, EE et al (2014) | Sweden | Swedish Medical Birth Register | 1996-2007 | Early neonatal Late neonatal Post-neonatal Infant | Narrative | Odds ratio | NA | Multiple logistic regression; Kruskal-Wallis/Wilcoxon tests |
| Stephenson, R et al (2017) | 38 low- and middle-income countries | Demographic and Health Survey | 2010+ | Neonatal Post-neonatal Infant Child | Narrative | Ratio of categories of twins | NA | Multilevel logistic regression |
| Subramanian, SV et al (2006) | India | Demographic and Health Survey | 1998-1999 (deaths in the two years preceding the interview) | Infant 2-5 years | Narrative | F:M sex ratio | NA | Multilevel logistic regression |
| Sudha, S and Rajan, SI (1999) | India | Census | 1981 and 1991 | Under-five | Narrative | M:F ratio | NA | Magnitude of sex ratios over time |
| Sudha, S and Rajan, SI (2003) | India | Census | 1981 and 1991 | Child | Narrative | sex ratio | NA | Sex ratio above or below one |
| Svedberg, P (1991) | 23 sub-Saharan African countries | Published estimates | 1953-1983 | Infant Child | Reference | M:F ratio | Sweden (1983-1987) | Excess male mortality = observed sex ratio / expected sex ratio (from reference population) |
| Tabutin, D (1978) | France, Canada, England and Wales, Belgium, Sweden | Official statistics report, published estimates | 1740-1939 | Infant Child | Narrative | M:F ratio | NA | Magnitude of sex ratios |
| Tabutin, D (1992) | Algeria, Morocco, Tunise, Egypt | Multiple sources | 1965-1988 | Infant Child | Comparative, not standard | M:F ratio | United Nations model life tables for developing countries | Magnitude of sex ratios |
| Tabutin, D and Willems, M (1995) | Sub-Saharan Africa | Demographic and Health Survey and World Fertility Survey | 1970s and 1980s | Infant Child Under-five | Narrative | M:F ratio | NA | Sex ratio above or below one |
| Tabutin, D and Willems, M (1998) | 24 countries (the whole of Europe, including Russia, Canada and the United States of America, Australia, New Zealand and Japan) | Literature, historical demographic statistics, specialists, statistical offices and research centres | 1600-1930 | Infant Child | Narrative | M:F ratio | NA | Magnitude of sex ratios |
| Tabutin, D et al (2001) | 38 African countries | National surveys | 1970-97 | Neonatal Post-neonatal Infant Child | Narrative | M:F ratio | NA | Sex ratio above or below one; overmortality level (difference between 100 and the ratio) |
| Takahashi, E (1954) | Japan | Vital statistics | 1941-1949 | Neonatal | Narrative | M:F ratio | NA | Magnitude of sex ratios |
| Tarozzi, A (2012) | India | National Family Health Survey | 1992-1993, 1998-1999 and 2005-2006 | Neonatal Infant Child Under-five | Narrative | F:M ratio | NA | Magnitude of sex ratios over time |
| Teitelbaum, MS (1971) | Scotland, Denmark, Swede, England and Wales, and Norway | Unpublished data from US National Center for Health Statistics | 1901-1963 | Early neonatal Perinatal | Narrative | Relative male risk of death M:F sex ratio | NA | Magnitude of sex ratios |
| Timaus, I et al (1998) | 41 low- and middle-income countries | Demographic and Health Survey | 1986-1994 (births in the 10 years preceding the interview) | Infant Child | Narrative | sex-specific mortality M:F ratio | NA | Magnitude of sex ratios |
| Ueyama, M (2007) | 55 low- and middle-income countries | Demographic and Health Survey | 1970-2000? | Under-five | Narrative | F:M ratio F-M difference | NA | Magnitude of sex ratios |
| United Nations (2011) | 149 countries | Vital registration, demographic surveys, and censuses | 1970s-2000s | Infant Child Under-five | Narrative | M:F ratio | NA | Magnitude of sex ratios |
| United Nations Secretariat (1998) | 88 countries with a population of one million or more in 1990 | Multiple sources | 1970-1988 | Infant Child Under-five | Narrative | M:F ratio | NA | Magnitude of sex ratios |
| Wahab, A et al (2001) | Purworejo District, Indonesia | Surveillance system | 1995-1996 | Infant | Narrative | M:F odds ratio Hazard ratios for each sex | NA | Cox proportional hazard models |
| Waldron, I (1987) | Developing countries | United Nations life tables and World Fertility Survey | 1964-1980 | Infant Child | Narrative | M:F ratio | NA | Magnitude of sex ratios |
| Wyllie, J (1933) | Canada | Official statistics | 1921-1930 | Neonatal Infant | Narrative | M:F ratio | NA | Magnitude of sex ratios |
| Xu, BH et al (1994) | China and Hong Kong | 10 Percent Sampling Tabulation on the 1990 Population Census; 10 Percent Sampling Tabulation on the 1982 Population Census; China's Cancer Epidemiology Survey of 1976; World Health Statistics Annual Series from Hong Kong | 1973-1975, 1981, 1990 | 0, 1, 2, 3, 4 years | Narrative | M-F difference M:F ratio | NA | Ratio and difference |
| Yount, KM (2001) | 14 Middle Eastern countries | United Nations | 1970s and 1980s | Infant Child Under-five | Comparative, standard | F:M ratio | Hill and Upchurch (1995) | Female disadvantage index |
| Yount, KM (2014) | 75 low- and middle-income countries | Demographic and Health Survey | 1985-2008 | Child | Narrative | F-M difference | NA | Multivariate models using fixed- and random effects approaches |
| Zhao, D et al (2017) | United States | United States National Center for Health Statistics | 1995-2004 | Perinatal Infant | Narrative | M:F odds ratio | NA | Conditional logistic regression |


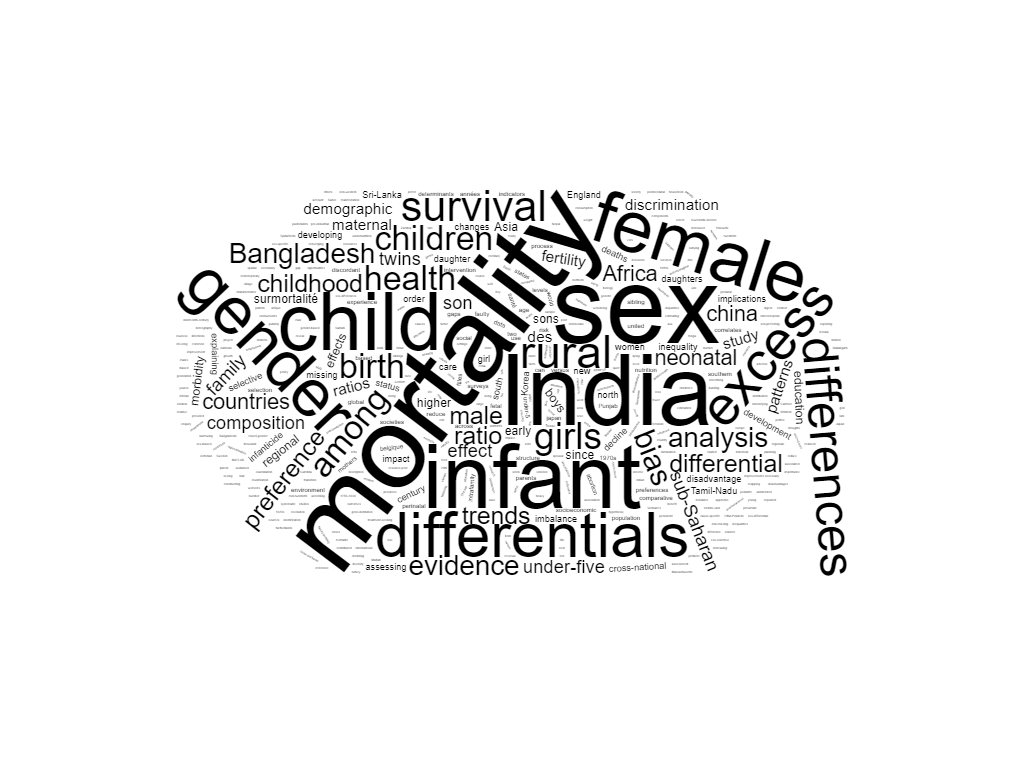


**Supplementary Figure S1 – Frequency of words used in the titles of the selected studies**
